# Identification and Characterization of a Male Sterile Rapeseed (*Brassica napus*) Line for Hybrid Seed Production

**DOI:** 10.3390/plants14091397

**Published:** 2025-05-06

**Authors:** Jianghua Shi, Huasheng Yu, Renhu Liu, Yaofeng Zhang, Ying Fu, Tanliu Wang, Xiyuan Ni, Tao Zheng, Jianyi Zhao

**Affiliations:** 1 Institute of Crop and Nuclear Technology Utilization, Zhejiang Academy of Agricultural Science, Hangzhou 310000, China; jhshi@zaas.ac.cn (J.S.); yuhuasheng-0@163.com (H.Y.); zhangy.f.zhang@163.com (Y.Z.); fy97@163.com (Y.F.); tanliuwtl@126.com (T.W.); jyzhao3@yahoo.com (J.Z.); 2 Institute of Biotechnology, Zhejiang Academy of Agricultural Science, Hangzhou 310000, China; liurh@zaas.ac.cn

**Keywords:** rapeseed (*Brassica napus* L.), genic male sterility, microspore development, BSA analysis, KASP marker

## Abstract

A male sterile mutant, S201, was identified in *Brassica napus*. Genetic analysis revealed that the male sterility trait was controlled by a recessive nuclear gene, *male sterility* (*MS*), which was stably inherited. The results of microscopy showed that the main reason for male sterility was a defect in microspore development, resulting in the absence of typical exine and mature microspores. Bulked segregant analysis (BSA) and genotyping of an F_2_ population showed that the *MS* gene was located in a 1.4 Mb region. Sequence analysis showed that the *CYP704B1* gene in this region contained two non-synonymous SNPs, leading to substitutions of two amino acids. A high-throughput KASP marker was characterized to detect the presence of the *ms* gene in the breeding population. The data presented here indicate that the male sterile mutant S201 can be applied in rapeseed breeding by producing the male sterile line and that the KASP marker developed for male sterility will be useful in marker-assisted selection of male sterile individuals in rapeseed-breeding programs.

## 1. Introduction

Rapeseed (*Brassica napus*; AACC, n = 38) is one of the most important oil crops worldwide. Rapeseed oil is used not only as an edible oil but also as an important raw material for biofuel and other industrial products [1,2]. The worldwide production of rapeseed increased from 70.99 million metric tons in the 2014–2015 season to 88.83 million metric tons in the 2022–2023 season [USDA ERS, 2024; https://www.ers.usda.gov/data-products/oil-cropsyearbook/] (accessed on 10 March 2025). The development and application of heterosis significantly contributes to this rapid increase in rapeseed production.

Heterosis (hybrid vigor) describes the superior performance of a hybrid generation as compared to its two parental varieties. Heterosis for inbreeding species (that is, species that usually self-pollinate) can offer between 20% and ≥50% yield increases [3]. Strategies for using heterosis are widely accepted for increasing yields in inbreeding crops, such as rice, wheat and rapeseed [3,4,5,6]. Male sterility is the most effective and commercially significant means of exploiting heterosis [5]. Several male sterility systems have been used in China, including cytoplasmic male sterility (CMS), cytoplasmic-induced male sterility (CIMS), self-incompatibility (SI), chemical hybridization agent (CHA), and genetic male sterility (GMS). Among these, cytoplasmic male sterility is widely utilized due to the ease of identifying maintainers in hybrid breeding [7,8,9,10]. However, CMS systems have common disadvantages, such as sensitivity to low temperature, incomplete sterility and limited availability of restorers [6,11,12].

Compared to CMS systems, GMS systems offer stable and complete male sterility, a broad range of restorer lines and minimal negative cytoplasmic effects. The double recessive two-type line system, such as S45AB, 117AB and Mian9AB, etc., is an important genic male sterile system in China [13,14,15]. Many hybrids, including the Youyan, Shuza, Deyou and Mianyou series, have been developed based on this system [16]. Another significant genic male sterile system is the recessive epistatic three-type line system, such as 7365ABC and the natural male sterility Lembke, which is widely used in China and Europe [5,17,18,19,20]. This highly effective three-line breeding system has led to the release of numerous commercial hybrids [5]. Despite these advantages, all GMS systems share a common drawback: half of the progeny of maternal parents are fertile, necessitating intensive labor to eliminate the male fertile plants [19,20]. This issue can be solved to some extent using high-throughput molecular markers, especially KASP (Kompetitive allele-specific PCR) markers, which facilitate the identification of different genotypes through genotyping.

In this study, we will examine the developmental defects of a male sterile rapeseed mutant (S201) by genetic analysis and microscopic observation. We cloned the *male sterility* (*MS*) gene by BSA analysis and map-based cloning and developed a KASP marker to detect the presence of the *ms* gene in the breeding population.

## 2. Results

### 2.1. Phenotypic and Genetic Analysis of the S201 Mutant

**Figure 1:**
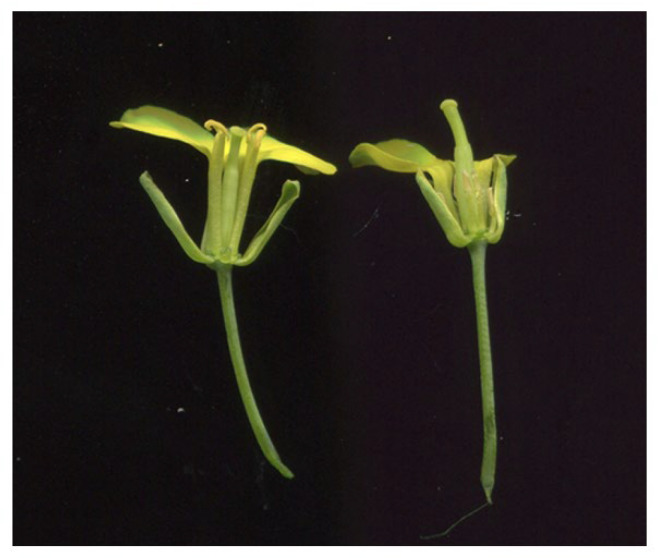
The flower phenotype of ZY50 (left) and S201 mutant (right). A portion of the corolla of each flower was removed to show the stamen morphology more clearly.

The rapeseed mutant S201 showed normal vegetative and floral development; however, it was completely male sterile. When compared to ZY50, the anthers from S201 were small, thin and pale yellow and S201 filaments were significantly reduced in length (Figure 1).

To understand the genetic behavior of the S201 mutant, crosses were made between S201 and two elite varieties ZY50 and ZS72, and F2 populations were developed from these two crosses. All the F_1_ plants from the two crosses were fertile (Table 1). In the F_2_ population derived from the cross between S201 and ZY50, the plants showed a segregation of 89 male fertile and 26 male sterile plants, displaying a ratio close to 3:1 (Table 1). In the F_2_ population derived from the cross between S201 and ZS72, the plants showed a segregation of 112 male fertile and 32 male sterile plants, also displaying a ratio close to 3:1 (Table 1). These results indicate that the male sterility phenotype is controlled by a single recessive gene.

### 2.2. The S201 Mutant Shows a Defect in Microspore Development

**Figure 2:**
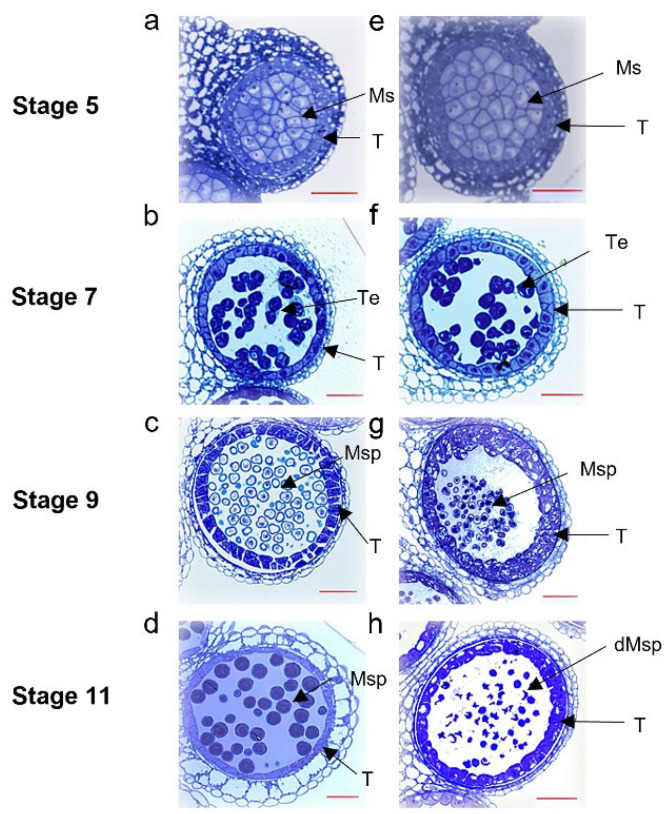
Cell biological analyses of wild-type and S201 mutant anthers. (**a**–**d**), wild-type (WT) anther morphology using semi-thin transverse sections. (**e**–**h**), morphology of S201 anthers using semi-thin transverse sections. Ms, microsporocytes; Msp, microspores; T, tapetal layer; Te, tetrads; dMsp, degenerated microspores. Bar = 50 μm.

Semi-thin section analysis was performed to examine morphological variations in anther development between S201 and ZY50. At stage 5 of anther development, there were no notable morphological differences between S201 and ZY50 (Figure 2a,e). However, at stage 7, the microspores in S201 appeared less regular, and more circular than those in ZY50 (Figure 2b,f). At stage 9, S201 showed an abnormally hypertrophic and vacuolated tapetum compared with that of ZY50 (Figure 2c,g). The microspores in S201 exhibited morphological abnormalities compared to those in ZY50 (Figure 2c,g). At stage 11, mature microspores were observed in ZY50, whereas the microspores in S201 had almost completely degenerated and collapsed (Figure 2d,h). Additionally, the tapetum in S201 remained in a hypertrophic and vacuolated state (Figure 2d,h).

**Figure 3:**
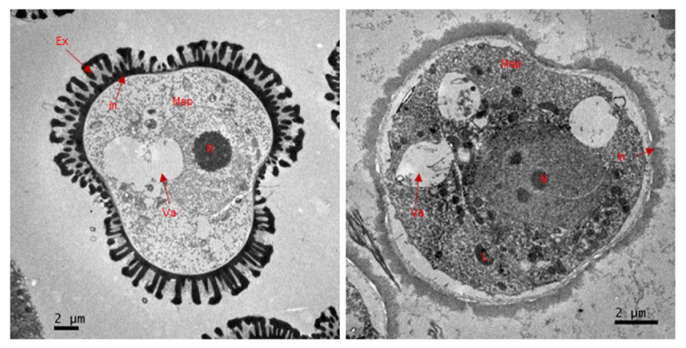
TEM micrographs of the anthers from the wild-type and the S201 mutant at stage 9. Ex, exine; In, intine; N, nucleolus; Msp, microspore; L, lipid body; Va, vacuole. Bar = 2 μm.

To further investigate the microspore defects in S201, transmission electron microscopy (TEM) was used to compare the ultrastructure of microspores between S201 and ZY50. At stage 9, the microspores in S201 had thinner walls and lacked the typical exine compared to ZY50 (Figure 3). The microspores also began to show some vacuolation (Figure 3). These findings collectively demonstrate that the S201 mutant fails to develop normal microspores at the flower stage, resulting in male sterility.

### 2.3. BSA and Genetic Mapping

After resequencing, raw read counts were obtained from four different bulks: ZY50 bulk (277,135,702 reads), S201 bulk (268,068,000 reads), F2 fertile bulk (304,007,462 reads), and F_2_ sterile bulk (228,195,740 reads). After quality control, over 95% of high-quality reads from all four bulks were uniquely mapped to the rapeseed reference genome (ZS11), revealing many single-nucleotide polymorphism (SNP) loci and insertion–deletion (InDel) loci. A total of 11,077,375 SNPs were identified between the male fertile bulk and male sterile bulks. The Δ(SNP-index) was calculated based on the SNP-index of the male fertile and male sterile bulks.

**Figure 4:**
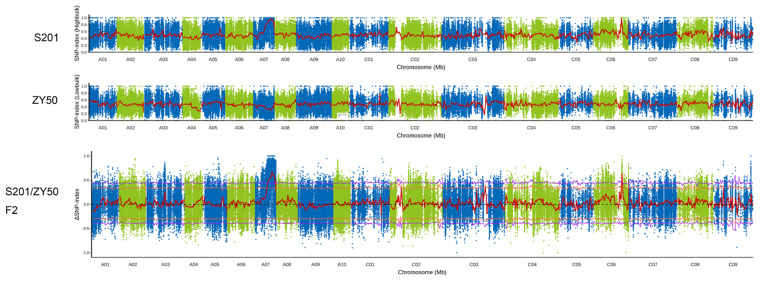
Manhattan plot of the distribution of SNP-index on chromosome in S201 and ZY50, and ΔSNP-index on chromosome in S201/ZY50 F2. The green dots and blue dots represent the values of SNP-index or of ΔSNP-index, respectively. The red line is the fit line of the SNP-index or ΔSNP-index by sliding-window analysis. The orange yellow line represents the 95% confidence interval upper side; the purple line represents the 99% confidence interval upper side.

Δ(SNP-index) graphs were generated, with the red line representing the confidence value (99%) (Figure 4). At this significance level, regions where the statistical confidence intervals of Δ(SNP-index) significantly deviated from 0 were considered possible candidate regions for the target gene. Only one region on chromosome A7 was significantly different from 0, spanning from 16,309,893 to 26,813,523 bp. These results suggest that the *male sterility* (*MS*) gene is located at this candidate region on chromosome A7 (Figure 4), which is also supported by high-density chip detection (Figure S1).

**Figure 5:**
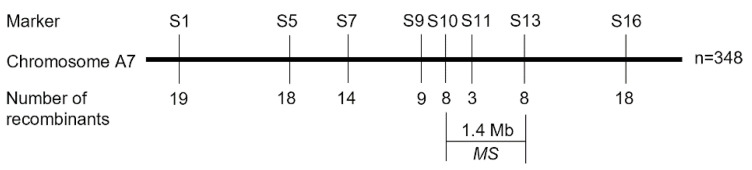
Map location of *MS* gene on rapeseed chromosome A7 in 348 F2 plants in which a region (1.4 Mb) is segregating. The thick bar represents the genomic region; the numbers underneath the bars indicate the number of recombinants between *MS* and the molecular marker.

Based on sequence data, eight SNP markers from the region spanning 16,309,893–26,813,523 bp were selected and tested for linkage to the *MS* gene in a population of 348 individuals from the F_2_ population (Figure 5). Two SNP markers, S10 and S13 each with eight recombinants, were found to be the closest flanking markers for the *MS* gene (Figure 5). According to the rapeseed genome sequence (ZS11), S10 and S13 are located 1.4 Mb apart. Detailed SNP marker information is provided in Table S1.

### 2.4. Sequence Analysis of the MS Gene

Comparison of this mapping region to the rapeseed reference genome (ZS11) showed that the *BnCYP704B1* gene on A7 chromosome was located in this mapping region, which was identified and named *BnMS1* in a prior study [13].

To identify the potential candidate gene for male sterility in S201, the open reading frames (ORFs) of *BnCYP704B1* were amplified from S201 and ZY50 and sequenced.

**Figure 6:**
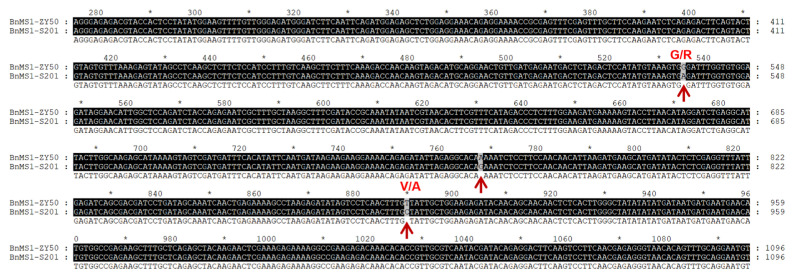
Alignment of partial nucleotide sequence of *MS* genes from ZY50 and S201. The red arrow represents point mutations occurred in the *MS* gene of S201. The red G/R and V/A represent amino acid substitutions due to SNPs in S201. The shading of the alignment presents as follows: identical residues in black and different residues in dark gray. “*” indicates positions which have a single, fully conserved residue.

Three SNPs at positions 535 (A535G), 768 (G768A) and 890 (C890T) from the translation start site were identified in the coding region of *BnCYP704B1* in S201 (Figure 6). Of these, the SNP at position 768 was a synonymous mutation that did not alter the amino acid. The two SNPs at position 535 and 890 in *BnCYP704B1* gene of S201 resulted in amino acid substitutions of G179R and V297A between ZY50 and S201 (Figure 6). Notably, the V297A substitution was reported to mutate the *BnCYP704B1* protein, resulting in male sterility in *Brassica napus* [13]. These findings suggest that the *MS* gene encodes the BnCYP704B1 protein, which is responsible for the male sterility phenotype in the S201 mutant.

### 2.5. Sequence Analysis of the MS Homolog

**Figure 7:**
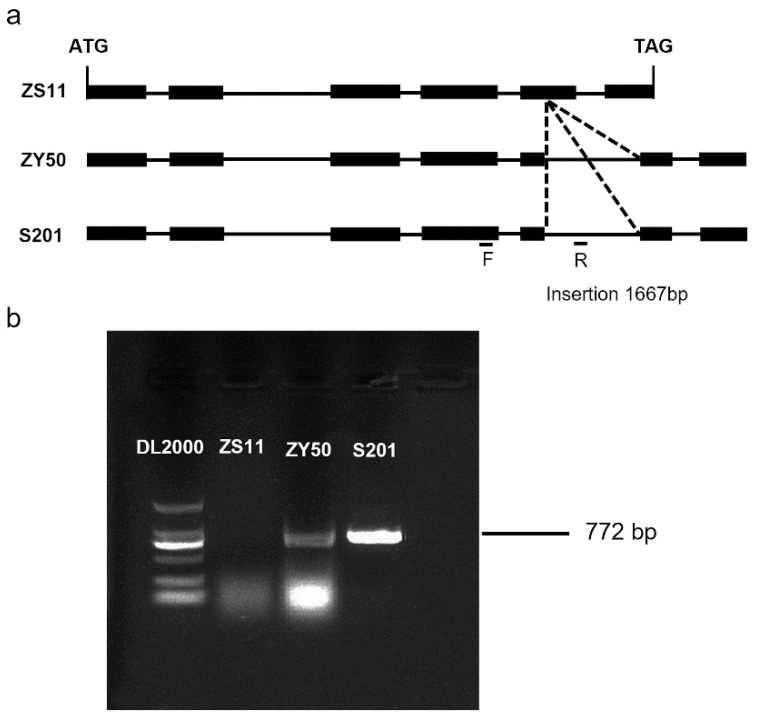
Structure characterization and RT-PCR analysis of *BnMS2* gene. (**a**) *BnMS2* structure and mutation site (fragment insertion). Exons are represented by filled black boxes and introns by lines. ATG and stop codon are shown with vertical lines. (**b**) RT-PCR analysis of *BnMS2* gene. The forward primer is located in the fourth exons and the reverse primer is located in the insertion fragment. Compared with ZS11, ZY50 and S201 included the insertion fragment.

A previous study reported that homologous genes of *BnCYP704B1* on A7 and C6 redundantly control male sterility in *Brassica napus* [13]. However, our results show that the male sterility phenotype of S201 is controlled by a single recessive gene. To explore this further, the *BnCYP704B1* homologous gene on C6, previously named *BnMS2* [13], was cloned and analyzed between ZY50 and S201. A comparison with the rapeseed reference genome (ZS11) showed that both S201 and ZY50 contained a 1667bp insertion in the fifth exon of the *MS* homolog (Figure S2). To further identify the fragment insertion in the *MS* homolog between ZY50 and S201, primers were specially designed, in which the forward primer was located in the fourth exon upstream of the insertion fragment and the reverse primer was located in the insertion fragment (Figure 7a). The RT-PCR results showed that the expected fragment, 772bp, could be amplified from S201 and ZY50, but not from the control cultivar, ZS11 (Figure 7b). A previous study showed that the fragment insertion in the fifth exon of the homolog resulted in the frameshift mutation, ultimately causing the protein to lose its function [13]. Our findings suggest that the *MS* homolog on C6 in both S201 and ZY50 was naturally mutated due to the fragment insertion in the fifth exon.

### 2.6. Genic Male Sterility Lines Selection in the Breeding Populations

In order to introduce the recessive *MS* gene into elite rapeseed cultivars, it was necessary to characterize and develop a high-throughput SNP marker for male sterility.

**Figure 8:**
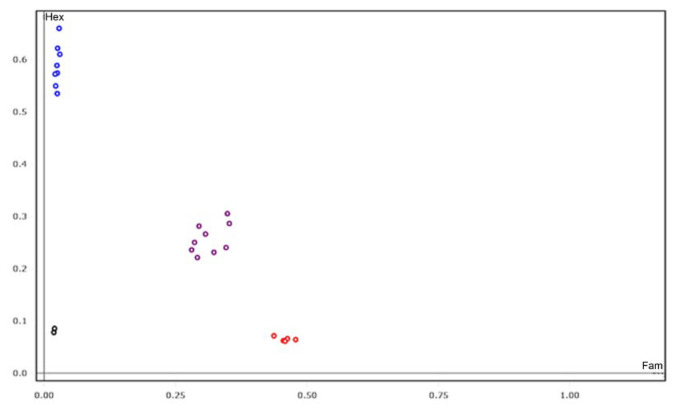
Kompetitive allele-specific PCR (KASPar) genotyping in a segregating rapeseed BC_1_F_2_ population for the selection of fertile-sterile lines. KASPar genotyping of *MS* gene on BC_1_F_2_ lines using functional marker S10. The blue, red, purple and black dots represent homozygous alleles (A/A; ZY50 genotype), homozygous alleles (G/G; S201 genotype), heterozygous alleles (A/G) and non-template control, respectively.

Based on our mapping results, the SNP marker S10 was selected to detect the presence of the *ms* gene due to its stableness and effectiveness (Figure 8). To confirm the KASP assay on male sterility, two distinct BC_1_F_2_ populations developed from ZY50/S201//ZY50 and ZS72/S201//ZS72 were genotyped using the S10 marker. The S10 marker resulted in three genotypes, as revealed by analysis of the seedlings in the BC_1_F_2_ populations (Table 2). The three genotypes are AA, Aa and aa, and the ratio of isolation of the three genotypes is 1:2:1 in the two distinct BC_1_F_2_ populations (Table 2). In the two BC_1_F_2_ populations developed from ZY50/S201//ZY50 and ZS72/S201//ZS72, 78 and 88 plants with AA and Aa genotypes showed male fertility; however, 24 and 19 homozygous plants with the genotype aa displayed male sterility (Table 2). The ratio of male fertility lines to male sterility lines is 3:1 in these two distinct BC_1_F_2_ populations (Table 2). Our results indicated that the genic male sterility lines could be easily and efficiently selected in the breeding populations using the S10 KASP marker.

## 3. Discussion

Male sterility is the most effective and commercially significant means of using heterosis, which helps to improve hybrid seed production, and to protect the commercial value of the parent lines. In this study, we identified a male sterile rapeseed mutant S201 (Figure 1). Genetic analysis showed that the male sterility trait in S201 is controlled by a single recessive nuclear gene (Table 1). The reason for male sterility is that S201 fails to develop normal pollen (Figure 2 and Figure 3). Using BSA analysis and sequence analysis, the *male sterility* (*MS*) gene in S201 was characterized as the *BnCYP704B1* gene on A7 (Figure 4, Figure 5 and Figure 6). Subsequently, an effective KASP marker was developed to detect the presence of the *ms* gene in the breeding population (Table 2). In summary, our results demonstrate that the S201 mutant and the related KASP marker can be used in rapeseed breeding to develop elite sterile lines.

CYP704B1 encodes an enzyme catalyzing the *ω*-hydroxylation of long-chain fatty acids, which belongs to an ancient and conserved family among terrestrial plants [4,21,22]. Cyp704B1 was reported to be involved in sporopollenin synthesis and exine formation in previous studies [20,21]. Previous studies on the rice cyp704b2 mutant [4], the rapeseed cyp704b1 mutant (S45A) [13] and the bread wheat triple cyp704b1 mutant [23] all demonstrate defects in sporopollenin synthesis and exine formation, resulting in a complete male sterile phenotype. In our study, the S201 mutant completely lost the normal exine and exhibited complete male sterility due to the mutation of the *CYP704B1* gene (Figure 1 and Figure 3). In Arabidopsis, the cyp704b1 mutant lacks a normal exine layer and has a characteristic striped surface, termed the zebra phenotype. Despite these structural changes, it shows a male fertile phenotype [21]. These results suggest that the effects of CYP704B on male sterility vary in distinct higher plants. The tapetum is a highly active secretory tissue, which plays an important role in the biosynthesis and secretion of maternally derived pollen wall components [24]. Cyp704B was also reported to be required for basic tapetal cell development and function [4,13]. For example, the rice cyp704b2 mutant showed a swollen sporophytic tapetal layer, aborted pollen grains without detectable exine, and undeveloped anther cuticle [4]. The rapeseed cyp704b1 mutant (S45A) exhibited abnormal tapetum development, defective exine, and aborted microspores due to the disturbed lipid metabolism [13]. In our study, the S201 mutant displayed a hypertrophic and vacuolated tapetum, lost exine, and produced aborted microspores (Figure 2 and Figure 3). Collectively, our results demonstrate that the mutated CYP704B1 caused complete male sterility in the S201 mutant through abnormal tapetum development and defective exine formation.

*BnCYP704B1* contains function-redundant double genes: the *MS* gene on chromosome A7 (*BnMS1*) and the *MS* homolog on C6 (*BnMS2*) in rapeseed. The S45A, 117A, Mian9A and S201 are all rapeseed cyp704b1 mutants, which suggests that the two *CYP704B1* genes are conserved genetic loci in rapeseed. In this study, two elite rapeseed cultivars in Zhejiang China, ZY50 and ZS72, were used as fertile parents to develop segregation populations (F_2_ or BC_1_F_2_) with the S201 mutant. Sequence analysis showed that ZY50 contained naturally mutated *BnMS2* genes (Figure 7 and Figure S2), indicating that *BnMS2* mutation is a common phenomenon in some released commercial cultivars. Taken together, these results suggest that the male sterility phenotype of S201 is controlled by a single recessive gene by analyzing the segregation population developed in this study.

As a genic male sterile mutant, S201 can be used to develop the male sterile line by introducing the recessive *ms* gene into elite rapeseed cultivars. The male sterile line has a commercial advantage because it is able to produce crossbred progenies with complete male sterility for hybrid seed production. In the GMS system, half of the progeny of maternal parents are fertile, requiring intensive labor to eliminate the male fertile plants from the maternal parents. A high-throughput SNP marker for male sterility should be characterized and developed to efficiently select genic male sterile individuals. In this study, eight SNPs and eight KASP markers associated with male sterility in *B. napus* were developed (Figure 5). KASP marker S10 was predominantly recommended for marker-assisted selection due to its stableness and effectiveness (Figure 8). Our results indicate that S201 can be applied in rapeseed breeding by producing male sterile lines and that the developed KASP marker is valuable for the high-throughput selection of male sterile individuals in rapeseed-breeding programs.

## 4. Materials and Methods

### 4.1. Plant Materials

The male sterility mutant (S201) used in this study was isolated from the breeding line QG 8-32 in a rapeseed experimental field. Two elite rapeseed cultivars ZY50 and ZS72 were used to develop segregation populations with S201. The cultivars ZS11, ZY50, ZS72 and S201 were used to sequence and compare the *male sterility* (*MS*) gene. The seeds of all materials were sown in the experimental fields of Zhejiang Academy of agricultural sciences, Hangzhou, China, in the winter–spring growing seasons. Approximately twenty plants were grown 20 cm apart in each row, with 30 cm between rows.

### 4.2. Inheritance Analysis

S201 male sterile plants were tagged and hand-pollinated with pollen from fertile ZY50 and ZS72 plants to generate two F_1_ generations. The F_1_ plants were then self-pollinated to harvest F_2_ generations. The fertility of F_1_ generations and the segregation ratio of fertility and sterility of F_2_ plants were measured at the flowering stage. The segregation of each population was tested by a Chi squared (*χ*^2^) goodness-of-fit test.

### 4.3. Light and Electron Microscopy

Fresh flower buds from both S201 and ZY50 plants were fixed in FAA (50% ethanol, 5% glacial acetic acid and 5% formaldehyde) overnight at 4 °C. Fixed flower buds were dehydrated using a graded ethanol series and embedded in Spurr resin. Transverse sections approximately 2 µm thick were cut from the embedded blocks using a Leica Ultracut R Ultramicrotome (Leica), Wetzlar, Germany. The sections were stained with a 2% toluidine blue O (Sigma-Aldrich, St. Louis, MO, USA) solution for cytological observation. Sections were photographed under a Zeiss Axiovert 200 microscope with a color CCD camera (Zeiss, Auberkheim, Germany).

To investigate the status of anthers development, anthers from S201 and ZY50 flower buds were prepared for transmission electron microscopy. The samples were vacuum-infiltrated and fixed in 2.5% glutaraldehyde in phosphate buffer (0.1 M, pH 7.2) for 4 h at 4 °C, rinsed, and incubated in 1% OsO4 in 0.1 M sodium phosphate buffer (pH 7.2) overnight at 4 °C. The samples were then rinsed again in phosphate buffer (0.1 M, pH 7.2), dehydrated in an ethanol series, infiltrated with a graded series of Spurr resin in acetone, and then embedded in Spurr resin. Thin sections were obtained using a diamond knife and a Reichert OM2 ultramicrotome (Buffalo, New York, NY, USA), and then stained in 2% uranyl acetate (pH 5.0), followed by 10 mM lead citrate (pH 12), and viewed with a transmission electron microscope (JEM-1230; JEOL, Sado city, Tokyo, Japan).

### 4.4. Bulked Segregant Analysis

F_2_ generation developed from S201 and ZY50 was used for bulked segregant analysis. Four DNA pools were constructed: the P_1_ pool from the S201 mutants, the P_2_ pool from ZY50, the male fertile pool from the male fertile plants of the F_2_ generation, and the male sterile pool from the male sterile plants of the F_2_ generation. These DNA pools were sequenced using the Illumina HiSeq 2500 platform with paired-end reads of 100 bp (Illumina, San Diogo, CA, USA).

Raw sequence reads from the four DNA pools were filtered and aligned to the rapeseed genome sequence (ZS11) using the Burrows–Wheeler alignment tool (BWA) [25,26]. GATK software (V3.5) was used to detect SNPs and InDels [27]. The candidate genomic regions associated with male sterility were identified by calculating the SNP-index and Δ(SNP-index) values [28]. The SNP-index was calculated based on the proportion of reads containing mutant parental genotypes in the reads covering a particular site on the genome in one bulk. The Δ(SNP-index) was determined based on the difference in the SNP-index between the male fertile and male sterile pools. An average of Δ(SNP-index) of SNPs located in the given genomic interval was calculated using a sliding window approach with a window size of 2 × 10^6^ Mb and 1 × 10^5^ kb increments.

The Δ(SNP-index) of male fertile and male sterile pools, along with their corresponding SNP-index within the specified window size, was plotted in a graph to generate SNP-index plots. The statistical confidence intervals of the Δ(SNP-index) value should be significantly distinct from 0 if a plotted region harbors the target gene [29]. The Δ(SNP-index) was calculated for all the SNP positions with given read depths, and 99% confidence intervals were obtained. The plot intervals above the significant threshold (99% confidence level) were considered candidate regions related to male sterility.

### 4.5. SNP Primer Design and KASP Genotyping

KASP genotyping was used to identify the SNP genotype in the segregation population and to construct a genetic map. According to the principle that the base type of the S201 pool was the same as the MS pool and the ZY50 pool was the same as the MF pool, all SNP loci obtained from sequence data were filtered and selected for primer design as described previously [12]. For each SNP, the KASP marker consisted of two SNP-specific primers and one common primer. Of these three primers, the SNP alleles were linked to the FAM and HEX fluorescent linker-specific sequence of the LGC KASP reagents at the 5′ end. The primer sequences are shown in Supplementary Table S1. SNP genotyping using an IntelliQube (LGC, Biosearch Technologies, London, UK) was conducted essentially as described previously [30]. The reaction conditions were as follows: 94 °C for 15 min, 10 touchdown cycles (94 °C for 20 s; touchdown at 61 °C, dropping to −0.6 °C per cycle 60 s), followed by 26 cycles of amplification (94 °C for 20 s, 55 °C for 60 s).

### 4.6. Cloning and Sequence Analysis of MS Alleles

Genomic DNA of young rapeseed leaves from S201, ZY50 and ZS11 was extracted with a modified cetyltriethylammnonium bromide (CTAB) method. Full-length *MS* was isolated and amplified separately from S201 and ZY50 using gene-specific primers. The allele was isolated and amplified separately from S201, ZY50 and ZS11 using the gene-specific primers listed in Supplementary Table S1. The resultant DNA fragments were purified and cloned into the pGEM-T Easy vector (Promega Corporation, Madison, WI, USA). The ligation vectors were then transformed into *E. coli* DH5α. Positive clones were sequenced by Tsingke Gene Company, Beijing, China. Nucleotide multiple-sequence alignment was constructed by use of the CLUSTAL OMEGA program [31] and visualized with the GeneDoc 3.2 program using the default BLOSUM scoring matrix.

### 4.7. RT-PCR Analysis

To analyze the characteristic of the *MS* homolog at the DNA level, a specific PCR primer pair was designed, with the forward primer located in the upstream sequence of the insertion fragment and the reverse primer located in the insertion fragment. Real-time PCR was conducted to confirm the resultant fragment, 772 bp. The primers used are listed in Supplementary Table S1.

### 4.8. Screening Male Sterility Plants in Rapeseed Breeding

BC_1_F_2_ populations developed from S201/ZY50//ZY50 and S201/ZS72//ZS72 were used for male sterility plant screening. The SNP markers used in gene mapping were screened for marker-assistant selection of the genic male sterility individuals in the breeding populations. KASP genotyping was carried out as described above.

## 5. Conclusions

Rapeseed mutant S201 exhibits complete male sterility due to a failure in microspore development. The *CYP704B1* gene is responsible for this male sterility trait in S201. S201 can be utilized in rapeseed breeding to produce the male sterile lines. Furthermore, the KASP marker developed for male sterility provides a valuable tool for high-throughput selection of male sterile individuals in rapeseed-breeding programs.

## Figures and Tables

**Table 1:** Phenotypic assessment for fertility in segregation populations.

Cross	Population	No. of Plants			*χ* ^2^	*p* Value
		Fertile	Sterile	Total		
S201 X ZY50	F_1_	45	0	45	_	_
	F_2_	89	26	115	0.350725	0.553703
S201 X ZS72	F_1_	52	0	52	_	_
	F_2_	112	32	144	0.592593	0.441418

**Table 2:** Validation of the KASP assays for fertility in distinct BC_1_F_2_ populations of *B. napus*.

Genotype	BC_1_F_2_ (No. of Plants)		Phenotype
	ZY50/S201//ZY50	ZS72/S201//ZS72	
AA	26	33	Fertile
Aa	52	50	Fertile
aa	24	19	sterile
*p* value (1:2:1)	0.995110034	0.143534986	-
χ^2^	0.00980392	3.882352941	-
-	0.731600589	0.137194216	*p* value (3:1)
-	0.11764706	2.209150327	χ^2^

A/a represents fertile/sterile allele for *BnMS* gene.

## Data Availability

All the data are presented in the manuscript, and further inquiries can be directed to the corresponding author.

## Supplementary Materials

The following supporting information can be downloaded at: https://www.mdpi.com/article/10.3390/plants14091397/s1, Figure S1. Genotyping analysis of ZY50, S201 and Fertile (F)/sterile (S) pools from F2 generation using high-density chip detection. Blue indicates the heterozygous genotype; gray represents homozygous genotype consistent with S201 and red represents homozygous genotype consistent with the parental ZY 50. Figure S2. Alignment of partial nucleotide sequence of the *MS2* gene from ZS11, ZY50 and S201. Sequence comparison shows that both ZY50 and S201 contain a 1667 bp fragment insertion at the 3′ end of 5′-TGGAGTACAACTGG-3′ in the 5th exon of *BnMS2* gene. Table S1. List of primers used in this study.

## Abbreviations

*MS*	Male sterility
CMS	Cytoplasmic male sterility
GMS	Genic male sterility
*B. napus*	*Brassica napus* L.
BSA	Bulked segregant analysis
RT-PCR	Reverse Transcription-Polymerase Chain Reaction
KASP	Kompetitive allele-specific PCR
