# Supplementary material for: Identification and Characterization of a Male Sterile Rapeseed (Brassica napus) Line for Hybrid Seed Production
Source: Plants (Basel). 2025 May 6;14(9):1397. doi: 10.3390/plants14091397 (PMC12073816; doi:10.3390/plants14091397)

## Slide 1
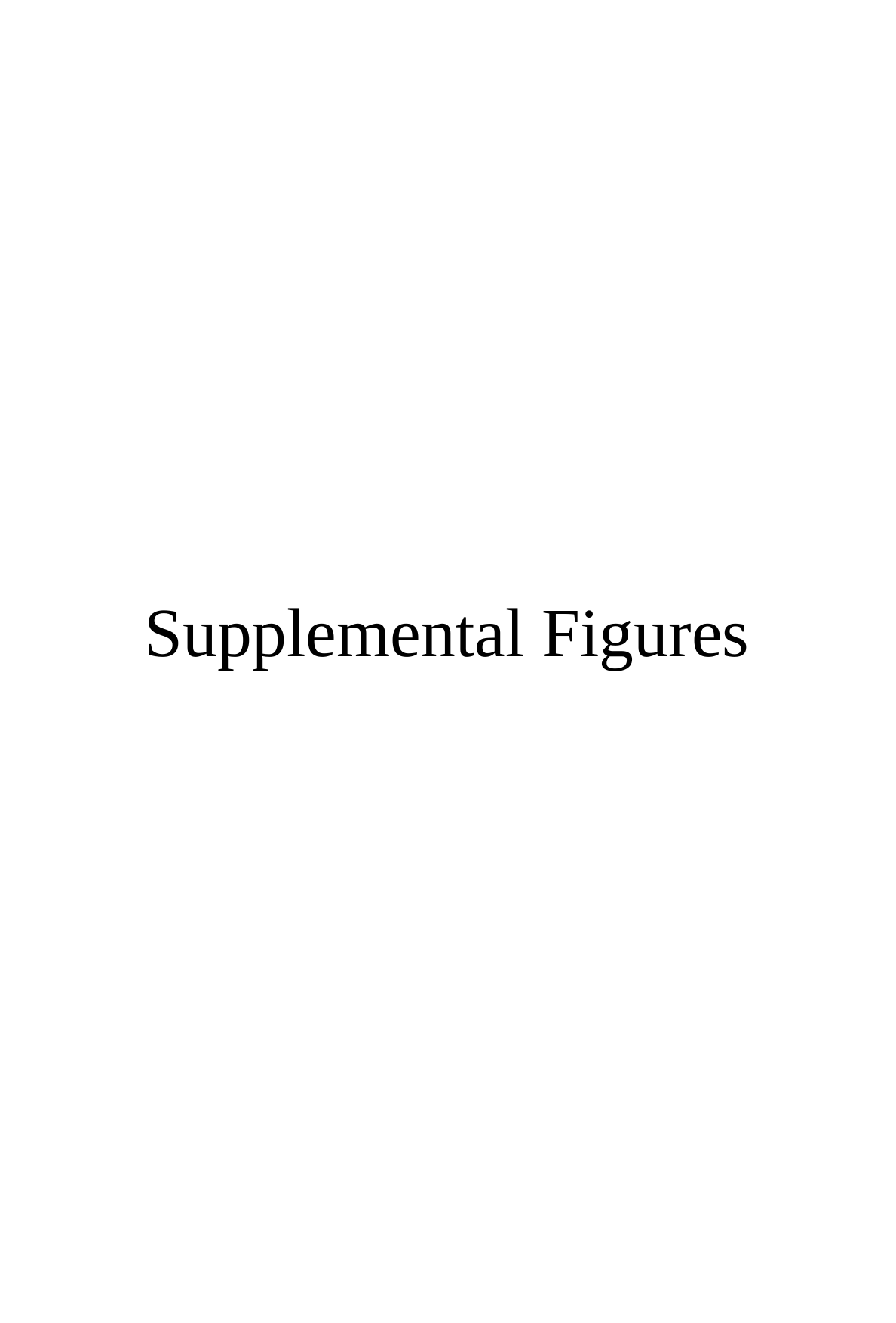

# Supplemental Figures

## Slide 2
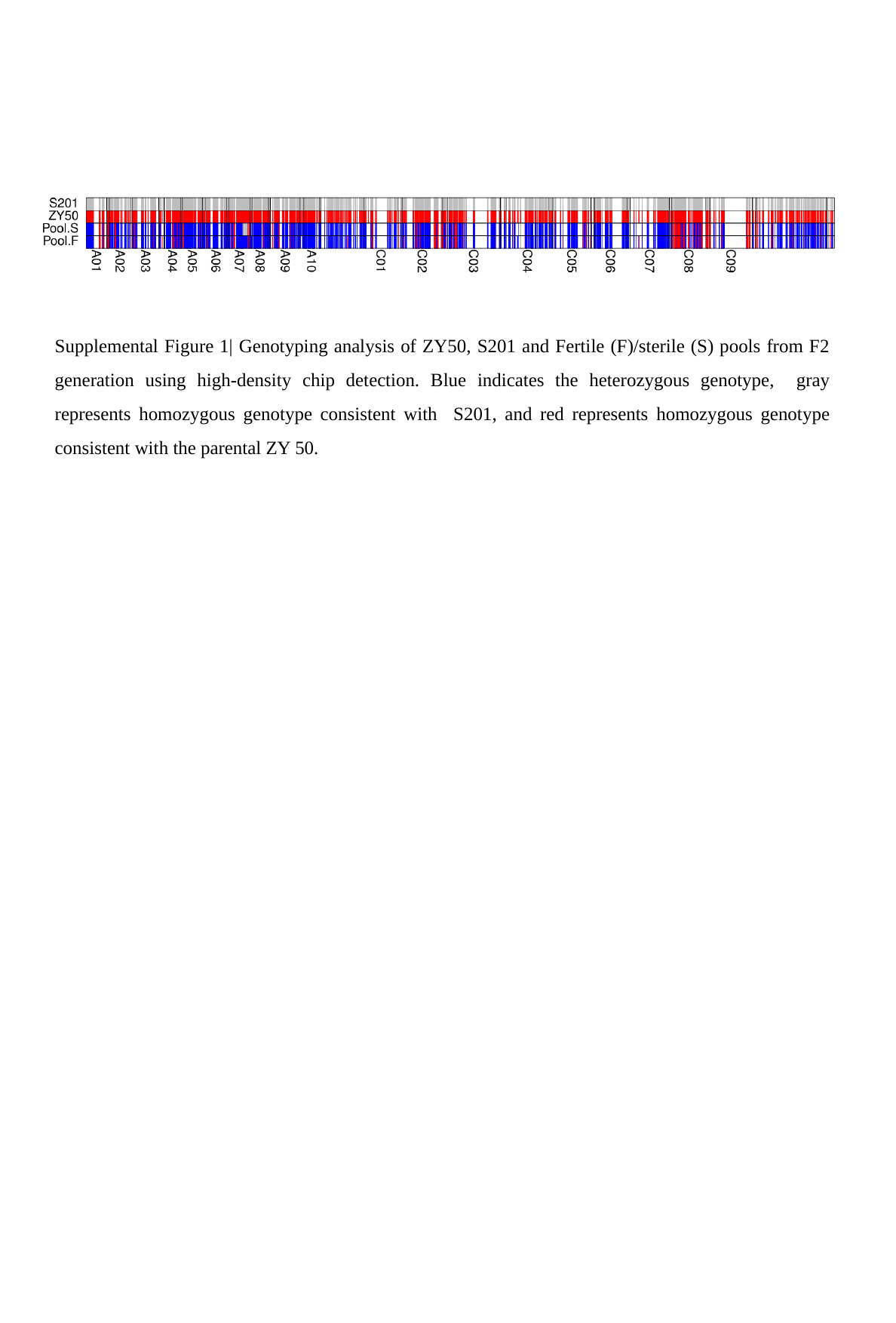

Supplemental Figure 1| Genotyping analysis of ZY50, S201 and Fertile (F)/sterile (S) pools from F2 generation using high-density chip detection. Blue indicates the heterozygous genotype, gray represents homozygous genotype consistent with S201, and red represents homozygous genotype consistent with the parental ZY 50.

## Slide 3
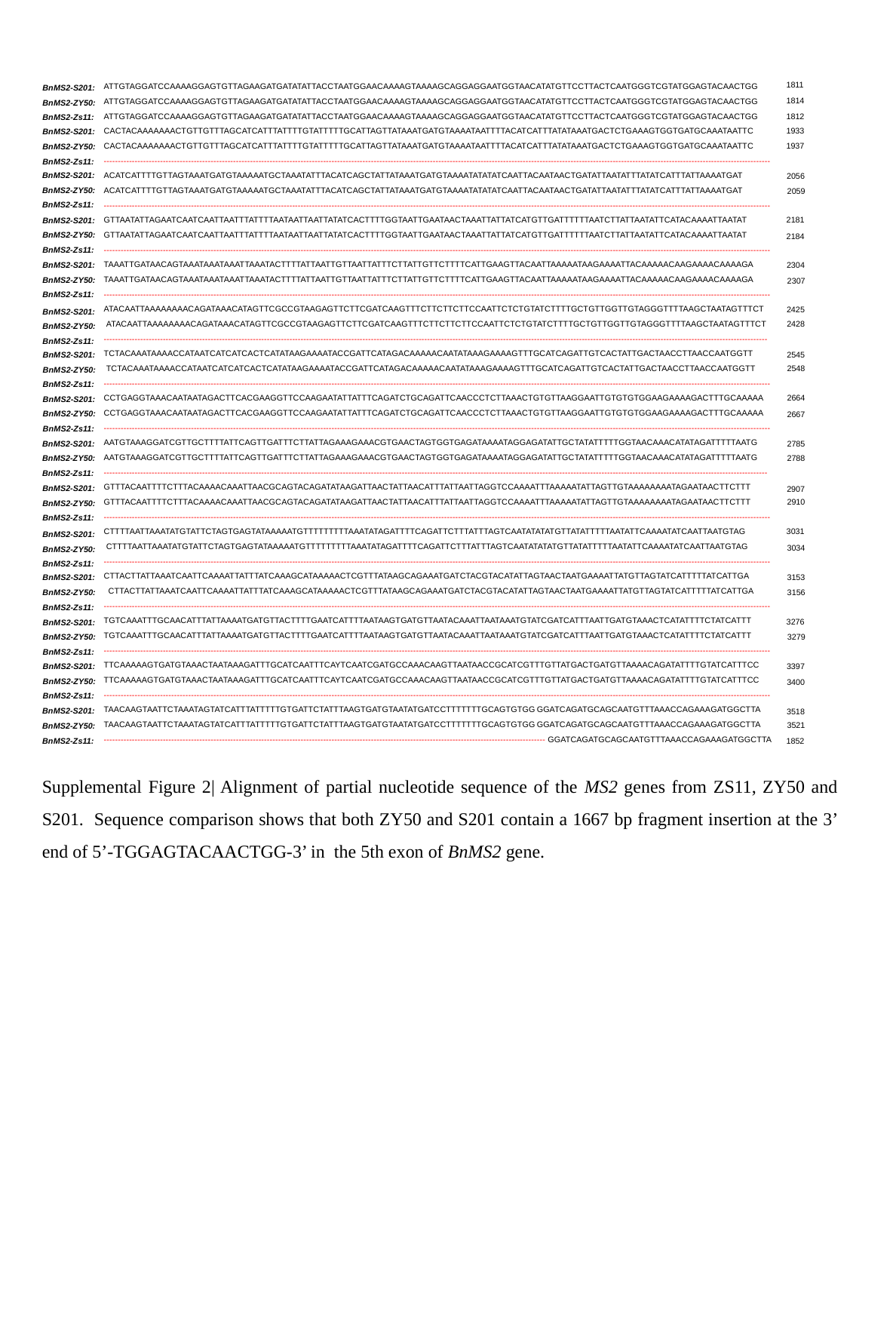

1811
Attgtaggatccaaaaggagtgttagaagatgatatattacctaatggaacaaaagtaaaagcaggaggaatggtaacatatgttccttactcaatgggtcgtatggagtacaactgg
Attgtaggatccaaaaggagtgttagaagatgatatattacctaatggaacaaaagtaaaagcaggaggaatggtaacatatgttccttactcaatgggtcgtatggagtacaactgg
Attgtaggatccaaaaggagtgttagaagatgatatattacctaatggaacaaaagtaaaagcaggaggaatggtaacatatgttccttactcaatgggtcgtatggagtacaactgg
CACTACAAAAAAACTGTTGTTTAGCATCATTTATTTTGTATTTTTGCATTAGTTATAAATGATGTAAAATAATTTTACATCATTTATATAAATGACTCTGAAAGTGGTGATGCAAATAATTC
CACTACAAAAAAACTGTTGTTTAGCATCATTTATTTTGTATTTTTGCATTAGTTATAAATGATGTAAAATAATTTTACATCATTTATATAAATGACTCTGAAAGTGGTGATGCAAATAATTC
---------------------------------------------------------------------------------------------------------------------------------------------------------------------------------------------------------------------------------------------
ACATCATTTTGTTAGTAAATGATGTAAAAATGCTAAATATTTACATCAGCTATTATAAATGATGTAAAATATATATCAATTACAATAACTGATATTAATATTTATATCATTTATTAAAATGAT
ACATCATTTTGTTAGTAAATGATGTAAAAATGCTAAATATTTACATCAGCTATTATAAATGATGTAAAATATATATCAATTACAATAACTGATATTAATATTTATATCATTTATTAAAATGAT
---------------------------------------------------------------------------------------------------------------------------------------------------------------------------------------------------------------------------------------------
GTTAATATTAGAATCAATCAATTAATTTATTTTAATAATTAATTATATCACTTTTGGTAATTGAATAACTAAATTATTATCATGTTGATTTTTTAATCTTATTAATATTCATACAAAATTAATAT
GTTAATATTAGAATCAATCAATTAATTTATTTTAATAATTAATTATATCACTTTTGGTAATTGAATAACTAAATTATTATCATGTTGATTTTTTAATCTTATTAATATTCATACAAAATTAATAT
---------------------------------------------------------------------------------------------------------------------------------------------------------------------------------------------------------------------------------------------
TAAATTGATAACAGTAAATAAATAAATTAAATACTTTTATTAATTGTTAATTATTTCTTATTGTTCTTTTCATTGAAGTTACAATTAAAAATAAGAAAATTACAAAAACAAGAAAACAAAAGA
TAAATTGATAACAGTAAATAAATAAATTAAATACTTTTATTAATTGTTAATTATTTCTTATTGTTCTTTTCATTGAAGTTACAATTAAAAATAAGAAAATTACAAAAACAAGAAAACAAAAGA
---------------------------------------------------------------------------------------------------------------------------------------------------------------------------------------------------------------------------------------------
ATACAATTAAAAAAAACAGATAAACATAGTTCGCCGTAAGAGTTCTTCGATCAAGTTTCTTCTTCTTCCAATTCTCTGTATCTTTTGCTGTTGGTTGTAGGGTTTTAAGCTAATAGTTTCT
 ATACAATTAAAAAAAACAGATAAACATAGTTCGCCGTAAGAGTTCTTCGATCAAGTTTCTTCTTCTTCCAATTCTCTGTATCTTTTGCTGTTGGTTGTAGGGTTTTAAGCTAATAGTTTCT
--------------------------------------------------------------------------------------------------------------------------------------------------------------------------------------------------------------------------------------------TCTACAAATAAAACCATAATCATCATCACTCATATAAGAAAATACCGATTCATAGACAAAAACAATATAAAGAAAAGTTTGCATCAGATTGTCACTATTGACTAACCTTAACCAATGGTT
 TCTACAAATAAAACCATAATCATCATCACTCATATAAGAAAATACCGATTCATAGACAAAAACAATATAAAGAAAAGTTTGCATCAGATTGTCACTATTGACTAACCTTAACCAATGGTT
---------------------------------------------------------------------------------------------------------------------------------------------------------------------------------------------------------------------------------------------
CCTGAGGTAAACAATAATAGACTTCACGAAGGTTCCAAGAATATTATTTCAGATCTGCAGATTCAACCCTCTTAAACTGTGTTAAGGAATTGTGTGTGGAAGAAAAGACTTTGCAAAAA
CCTGAGGTAAACAATAATAGACTTCACGAAGGTTCCAAGAATATTATTTCAGATCTGCAGATTCAACCCTCTTAAACTGTGTTAAGGAATTGTGTGTGGAAGAAAAGACTTTGCAAAAA
---------------------------------------------------------------------------------------------------------------------------------------------------------------------------------------------------------------------------------------------
AATGTAAAGGATCGTTGCTTTTATTCAGTTGATTTCTTATTAGAAAGAAACGTGAACTAGTGGTGAGATAAAATAGGAGATATTGCTATATTTTTGGTAACAAACATATAGATTTTTAATG
AATGTAAAGGATCGTTGCTTTTATTCAGTTGATTTCTTATTAGAAAGAAACGTGAACTAGTGGTGAGATAAAATAGGAGATATTGCTATATTTTTGGTAACAAACATATAGATTTTTAATG
--------------------------------------------------------------------------------------------------------------------------------------------------------------------------------------------------------------------------------------------GTTTACAATTTTCTTTACAAAACAAATTAACGCAGTACAGATATAAGATTAACTATTAACATTTATTAATTAGGTCCAAAATTTAAAAATATTAGTTGTAAAAAAAATAGAATAACTTCTTT
GTTTACAATTTTCTTTACAAAACAAATTAACGCAGTACAGATATAAGATTAACTATTAACATTTATTAATTAGGTCCAAAATTTAAAAATATTAGTTGTAAAAAAAATAGAATAACTTCTTT
---------------------------------------------------------------------------------------------------------------------------------------------------------------------------------------------------------------------------------------------
CTTTTAATTAAATATGTATTCTAGTGAGTATAAAAATGTTTTTTTTTAAATATAGATTTTCAGATTCTTTATTTAGTCAATATATATGTTATATTTTTAATATTCAAAATATCAATTAATGTAG
 CTTTTAATTAAATATGTATTCTAGTGAGTATAAAAATGTTTTTTTTTAAATATAGATTTTCAGATTCTTTATTTAGTCAATATATATGTTATATTTTTAATATTCAAAATATCAATTAATGTAG
---------------------------------------------------------------------------------------------------------------------------------------------------------------------------------------------------------------------------------------------
CTTACTTATTAAATCAATTCAAAATTATTTATCAAAGCATAAAAACTCGTTTATAAGCAGAAATGATCTACGTACATATTAGTAACTAATGAAAATTATGTTAGTATCATTTTTATCATTGA
 CTTACTTATTAAATCAATTCAAAATTATTTATCAAAGCATAAAAACTCGTTTATAAGCAGAAATGATCTACGTACATATTAGTAACTAATGAAAATTATGTTAGTATCATTTTTATCATTGA
---------------------------------------------------------------------------------------------------------------------------------------------------------------------------------------------------------------------------------------------
TGTCAAATTTGCAACATTTATTAAAATGATGTTACTTTTGAATCATTTTAATAAGTGATGTTAATACAAATTAATAAATGTATCGATCATTTAATTGATGTAAACTCATATTTTCTATCATTT
TGTCAAATTTGCAACATTTATTAAAATGATGTTACTTTTGAATCATTTTAATAAGTGATGTTAATACAAATTAATAAATGTATCGATCATTTAATTGATGTAAACTCATATTTTCTATCATTT
---------------------------------------------------------------------------------------------------------------------------------------------------------------------------------------------------------------------------------------------
TTCAAAAAGTGATGTAAACTAATAAAGATTTGCATCAATTTCAYTCAATCGATGCCAAACAAGTTAATAACCGCATCGTTTGTTATGACTGATGTTAAAACAGATATTTTGTATCATTTCC
TTCAAAAAGTGATGTAAACTAATAAAGATTTGCATCAATTTCAYTCAATCGATGCCAAACAAGTTAATAACCGCATCGTTTGTTATGACTGATGTTAAAACAGATATTTTGTATCATTTCC
---------------------------------------------------------------------------------------------------------------------------------------------------------------------------------------------------------------------------------------------
TAACAAGTAATTCTAAATAGTATCATTTATTTTTGTGATTCTATTTAAGTGATGTAATATGATCCTTTTTTTGCAGTGTGGggatcagatgcagcaatgtttaaaccagaaagatggctta
TAACAAGTAATTCTAAATAGTATCATTTATTTTTGTGATTCTATTTAAGTGATGTAATATGATCCTTTTTTTGCAGTGTGGggatcagatgcagcaatgtttaaaccagaaagatggctta
-------------------------------------------------------------------------------------------------------------------------------------------------------------ggatcagatgcagcaatgtttaaaccagaaagatggcTTA
BnMS2-S201:
BnMS2-ZY50:
BnMS2-Zs11:
1814
1812
1933
BnMS2-S201:
BnMS2-ZY50:
BnMS2-Zs11:
1937
BnMS2-S201:
BnMS2-ZY50:
BnMS2-Zs11:
2056
2059
2181
BnMS2-S201:
BnMS2-ZY50:
BnMS2-Zs11:
2184
BnMS2-S201:
BnMS2-ZY50:
BnMS2-Zs11:
2304
2307
2425
BnMS2-S201:
BnMS2-ZY50:
BnMS2-Zs11:
2428
2545
BnMS2-S201:
BnMS2-ZY50:
BnMS2-Zs11:
2548
2664
BnMS2-S201:
BnMS2-ZY50:
BnMS2-Zs11:
2667
2785
BnMS2-S201:
BnMS2-ZY50:
BnMS2-Zs11:
2788
BnMS2-S201:
BnMS2-ZY50:
BnMS2-Zs11:
2907
2910
3031
BnMS2-S201:
BnMS2-ZY50:
BnMS2-Zs11:
3034
3153
BnMS2-S201:
BnMS2-ZY50:
BnMS2-Zs11:
3156
3276
BnMS2-S201:
BnMS2-ZY50:
BnMS2-Zs11:
3279
3397
BnMS2-S201:
BnMS2-ZY50:
BnMS2-Zs11:
3400
BnMS2-S201:
BnMS2-ZY50:
BnMS2-Zs11:
3518
3521
1852
Supplemental Figure 2| Alignment of partial nucleotide sequence of the MS2 genes from ZS11, ZY50 and S201. Sequence comparison shows that both ZY50 and S201 contain a 1667 bp fragment insertion at the 3’ end of 5’-TGGAGTACAACTGG-3’ in the 5th exon of BnMS2 gene.

## Slide 4
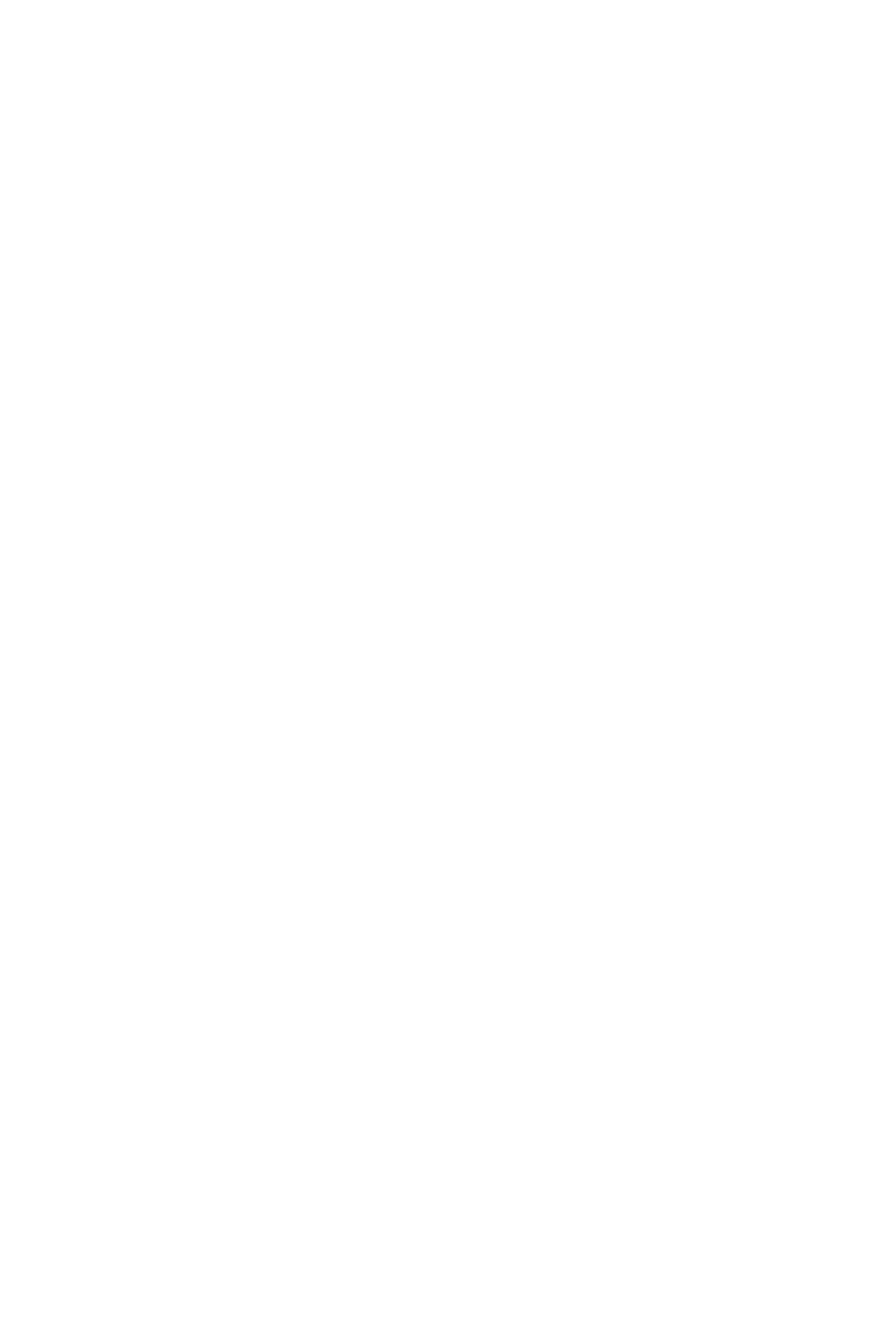

Supplement: Supplementary file 1 [file plants-14-01397-s001.zip › Supplemental Figures.pptx]
